# Comparison of Microwave Short Time and Oven Heating Pretreatment on Crystallization of Raisins

**DOI:** 10.3390/foods10010039

**Published:** 2020-12-25

**Authors:** Lorena Alvarez, Paulina Urrutia, Araceli Olivares, Agustín Flores, Bhesh Bhandari, Sergio Almonacid

**Affiliations:** 1 Centro Regional de Estudios en Alimentos Saludables (CREAS), 2373223 Valparaíso, Chile; lalvarez@crea.cl (L.A.); paulina.urrutia@gmail.com (P.U.); aflores@creas.cl (A.F.); sergio.almonacid@usm.cl (S.A.); 2 Escuela de Ingeniería Bioquímica, Facultad de Ingeniería, Pontificia Universidad Católica de Valparaíso, 2362803 Valparaíso, Chile; 3 School of Agriculture and Food Sciences, The University of Queensland, St. Lucia, QLD 4072, Australia; b.bhandari@uq.edu.au; 4 Departamento de Ingeniería Química y Ambiental, Universidad Técnica Federico Santa María, 2340000 Valparaíso, Chile

**Keywords:** Chilean raisin, crystallization, sugaring, temperature, relative humidity, microwave

## Abstract

Crystalline material can develop on the surface of raisins during storage and transport, affecting the final acceptability of the product. In this work, a mild thermal pretreatment was applied to raisins to melt the pre-existing crystals and the effect of such thermal treatments on the development of crystals over a storage period was investigated. The raisins selected for this study were of the Thompson seedless variety from one Chilean company. The thermal pretreatment of raisins at 50 °C and 70 °C for 20 min in an oven and microwave (800 W) irradiation for 15 s resulted in a reduction in the percentage of crystallized raisins (*w*/*w*) from more than 50% in the control samples to less than 10% after 35 days of storage at 15 and 25 °C in a 57% relative humidity environment. The results showed that some textural parameters, such as cohesiveness and chewiness, were not affected by thermal treatment and were independent of storage temperature.

## 1. Introduction

Consumption of raisins is widespread. They are an important source of nutrients, vitamins and minerals [1]. Being in dehydrated form, raisins are easily transported and shipped.

Through the season of 2017–2018, the global production of raisins was 1.149 million metric tons, mainly from Turkey, the United States, China, and Iran; together representing 73% of the world production [2]. Chile is another important producer, reaching 57,000 metric tons during the same season, according to the Chilean Department of Agriculture. Chilean raisins are principally exported (96.5%) [2] and their main markets are the United State, United Kingdom, Netherland, Russia, and Colombia [3].

The dehydration process to transform grapes into raisins entails many physical and chemical mechanisms [4]. Exposure to a high temperature and water loss during dehydration results in shrinkage, crystallization, and color, flavor and texture alterations of the final product (raisin). All these changes may adversely affect the quality of the dried product. In this context, recent studies have reported the effect of processing variations on the physical, chemical and phenolic composition of raisins [5]. In particular, during prolonged transport and storage of raisins, a visible crystalline material can habitually develop in its flesh and surface. The surface crystals harm the raisin’s appearance and texture, and make them to be likely rejected by the consumers [3,5,6,7]. The United State Standards indicates that for raisins of grade A, B, and C, the maximum (percent by weight) acceptable crystallized or sugared raisins (controlled by visual inspection) are 5, 10 and 15%, respectively [6]. The storage condition and time are the principal causes of sugar crystallization. Humid climates, fluctuating storage temperature as well as excessive handling and abrasion are the conditions that can promote the crystallization of sugars [7]. Raisins exported from Chilean producers have been reported to be seriously affected by the sugaring phenomena during shipping, as a consequence of fluctuating storage conditions in shipping and customer storage thereafter. Consequently, crystallization has been one of the main concerns for Chilean raisin producers.

Water activity (a_w_) and glass transition temperature (T_g_) have been normally used to explain the effects of environmental factors on the physicochemical changes associated with the food stability. It is well known that dehydrated food materials can exist in a “glassy state” that exhibits no or neglected mobility in a practical time scale, which keeps the structure of the material hard and brittle, or in a “rubber state”; liquid-like viscoelastic state which favors structural collapse. The temperature at which the glassy material system transforms to a rubbery state is called the glass transition temperature (T_g_). This depends on the moisture content of the material, as well as on its molecular nature [8]. The molecular mobility which promotes chemical and physical changes, is regained in the rubbery state. In this context, both, nucleation and the crystal growth rate are functions of molecular mobility; nucleation rate maximizes just above T_g_, while the crystal growth rate is the highest between T_g_ and T_m_ (equilibrium melting temperature) [9].

It has been reported that for sultana raisins at a_w_ of 0.536, the onset and midpoint of T_g_ are −32.28 °C and −28.39 °C, respectively, when using a scan rate of 10 °C min^−1^ [10]. This means that to keep a low crystal growth rate, so that product quality can be maintained during its shelf life period, the temperature must be kept under −32.28 °C, which is of low practical feasibility.

Recently, Truong et al. [11] investigated the crystallization and glass transition temperature (T_g_) as affected by the sugar component ratio, water fraction, and storage conditions on three Chilean raisins that were examined by means of differential scanning calorimetry (DSC), polarized light microscopy and X-ray diffraction (XRD) techniques. Measurement of T_g*s*_ for different water activities (a_w_ 0.11–0.74) indicated that water acts as an important plasticizer that reduced the T_g*s*_ values (−16.4 to −61.6 °C). XRD results revealed that Thompson raisins sugaring was retarded at low temperatures (5 °C and 15 °C) compared to those stored at 25 °C. This was attributed to the maximization of the nucleation but minimization of the crystal growth at the lower temperature.

In the earlier days, few attempts were made to address the sugaring problem in raisins. Bolin [12] found that if whole raisins are subjected to a mild heat pretreatment (49 °C for 48 h), the formation of a significant amount of crystals, observed by visual inspection, can be delayed over a period of 10 months of storage at 22 °C. The heating resulted in the melting of the existing crystals in the raisins, which otherwise would grow larger during the transport and storage periods and cause sugaring in the raisins. This heat pretreatment caused insignificant darkening of the raisins. Similarly, Akev et al. [13] studied the impact of low temperature storage (0 °C, 75–85% relative humidity) and found that raisins packaged under controlled atmosphere (1% O_2_ and 3% CO_2_) also resulted in no sugaring after 10 months, as evaluated by sensory analysis.

The above studies provide important information, nonetheless, the high temperature pretreatment has greater potential application, since it does not require the maintenance of a costly low temperature chain during the raisin shelf-life period. Under this consideration, Alvarez et al. [14] investigated the impact of high temperature pretreatment (50 °C for 12 or 48 h) on the sugaring phenomenon. They used a visual inspection technique along with X-ray diffraction and polarized light microscopy to observe and evaluate sugar crystals after pretreatment, and the sugaring rate and crystal size during storage at 15 °C and 25 °C. It was found that the thermal pretreatment reduced the fraction of crystallized raisins (*w*/*w*) from 46% (control) to less than 10%, after 30 days storage. Following this line of high temperature pretreatment, the application of microwave technology seems to be an attractive alternative source of thermal energy with the capability of rapidly heating the interior of the raisins, consequently melting the tiny existing crystals in the raisin flesh. Microwave heating has been reported to improve the general efficiency of food processing due to significant advantages over conventional heating methods. It has been used in many food processing lines such as cooking, pasteurization, sterilization, thawing, baking, blanching and drying processes, including those involving grapes [15,16].

Microwave pretreatment has been used as an alternative method (no chemicals) to loosen the natural waxy coating on grapes, which impedes water diffusion through the peel and delays moisture removal, with a significant impact on the drying time and resultant product quality [17,18]; a similar process was reported for drying cranberries [19]. The results showed that the microwave-treated samples had a significantly higher total soluble solids (TSS) content, along with good appearance and market quality. However, no published report has been found regarding the use of microwave pretreatment to control the sugaring phenomenon.

The present study aims to explore the potential advantages of applying microwave technology to volumetrically heat whole raisins as a pretreatment in order to melt the existing crystals that can grow in an uncontrolled way over the time, and to compare it with the conventional air-heating oven processing. Visual inspection and polarized light microscopy were used to measure the sugaring rate and to observe and determinate the sugar crystals size, when raisins were stored at two different temperatures in a given humidity condition. Additionally, texture and color analyses of pretreated samples were undertaken after one month of storage.

## 2. Materials and Methods

### 2.1. Materials

The raisins selected for this study were of the Thompson seedless variety, size jumbo (>12 mm), provided by the Chilean company Frutexsa, located in the Aconcagua Valley, Chile. The raisins were harvested and processed in the period between 2017–2019. Before being processed, dried grapes were stored in warehouses at uncontrolled temperature and humidity. These parameters were monitored throughout the season using temperature and relative humidity sensors. The temperature varied between 16–30 °C, while the relative humidity varied between 37–40% (from February to May).

The raisins used in this work were the finished product, ready for storage or commercialization. The tests were carried out with samples of no more than one month after processing.

All chemicals used in this study were of analytical grade unless otherwise specified.

### 2.2. Analyses on Chemical Composition and Physico-Chemical Properties of Initial Samples

The raisins samples were ground using a low speed grinder (IKA MF10 basic, Campinas, Brazil). The raisin paste was then subjected to determination of sugar composition, total soluble solids, pH, acidity, moisture content and water activity.

#### 2.2.1. Sugar Composition

Analysis of glucose and fructose in the initial raisin samples was undertaken using high-performance liquid chromatography (HPLC) with a refractive index detector (IR), JASCO brand (model C-4061, Tokyo, Japan). A Benson 1400-0BP-100Ag + Carbohydrate column (Benson Polymeric Inc., Reno, NV, USA) was used, with a flow rate of 0.35 mL/min at 55 °C, and detector temperature of 45 °C. Each run required 35 min to complete. Stock solutions of glucose and fructose (10% *w*/*w*) were prepared, whereby working standard solutions within the concentration ranges of 25–500 mg/L were prepared fresh. Quantification of sugar level was based on integrated peak areas as against the corresponding external standards. Samples were filtered using syringe filters (0.45 µm nylon).

#### 2.2.2. pH and Titrable Acidity

The ground raisin samples were diluted with distilled water to prepare 10% *w*/*w* raisin solution. pH was determined by using a pH meter (Jenco-pH meter, JENCO, CA, USA) according to Dehghan-Shoar et al. [20].

Distilled water was added to the ground raisins to make up a raisin solution (20% *w*/*w*). The raisin solution was mixed well and filtered through cheese cloth to remove the insoluble parts. Twenty-five mL of the filtered raisin solution was titrated with NaOH (0.1%) using phenolphthalein as an indicator. The titrable acidity of the raisin samples was expressed as percentage of anhydrous tartaric acid [21] using the following equation:

% dry weight = 0.1 × *N* × V_m_ × (75 gmol) × 25 mL × (100) (1)

in which *N* is normality of NaOH used, V_m_ is volume (mL) of NaOH consumed by titrant.

#### 2.2.3. Moisture Content and Water Activity

Approximately 2 g of the raisin ground samples was spread thinly onto the aluminum dishes, which were then allowed to dry under vacuum conditions using a vacuum drying oven (DZ-2B CII oven, Company, City, (Hyhoo Scientific supplies, Mundolab Ltda., Santiago, Chile) at 60 °C until reaching the constant weight. The moisture content was calculated based on the difference in weight before and after vacuum drying.

Determination of the water activity of the raisin ground samples was undertaken at 25 °C using a water activity meter (Aqua Lab meter, Decagon, WA, USA).

#### 2.2.4. Texture Profile Analysis (TPA)

**Figure 1:**
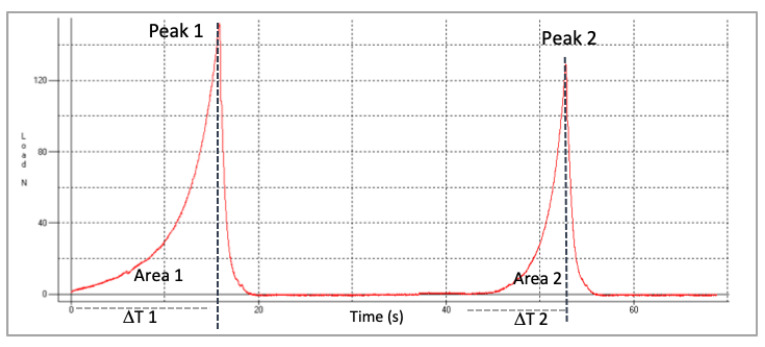
Representative Texture Profile Analysis (TPA) curve of fresh raisins. Peak 1 and Peak 2 are the maximum load or force (N) for first and second cycle of compression, respectively.

For the evaluation of the textural parameters the equipment (TMS-pro model, Food Technology Corp, West Sussex, UK) was used. Analysis was carried out using two compression cycles using a flat 45 mm diameter plunger. Interval between cycles was 5 s. An amount of 40 g of raisins (approximately 35 raisins) was used to load a cylindrical probe and the test rate was 100 mm/min. The compression used was 20%. Five texture profile analyses (TPAs) were obtained for each sample. Figure 1 represents the typical double compression or TPA test of fresh raisins (load or force/time).

The texture parameters of hardness, springiness (elasticity), cohesiveness and chewiness were calculated by the following equations based in definitions of TPA [22].

(2) $$\mathrm{Hardness}\;\left(N\right)=F_{1}$$

(3) $$\mathrm{Springiness}\;\left(\%\right)=\;\Delta T_{2}/\Delta T_{1}\;\times \;100$$

(4) $$\mathrm{Cohesiveness}\;\left(\mathrm{dimensionless}\right)={\;A}_{2}/A_{1}$$

(5) $$\mathrm{Chewiness}\;\left(N\right)={\;F}_{1}\;\times \;\Delta T_{2}/\Delta T_{1}\;\times {\;A}_{2}/A_{1}$$

where, T_1_, T_2_ = times; A_1_, A_2_ = Areas under the peaks.

#### 2.2.5. Color Attributes of Raisins

The color parameters of raisins were measured using a colorimeter (Konica MINOLTA CHROMA METER CR-400, Osaka, Japan) with and without thermal pretreatment of raisins after 30 days of incubation at different storage conditions (temperature and relative humidity). The values were expressed by the CIE L* (brightness–darkness), a* (+a*: red, −a*: green) and b* (+b*: yellow, −b*: blue) system. The values obtained for the L*, a* and b* parameters were the average of 20 samples examined.

ΔE, represents the total color difference and is considered for the overall color evaluation between fresh raisins (without pretreatment) and thermal pretreated samples (after 35 days). Raisins without thermal pretreatment or fresh raisins samples ($L_{0}^{*}$, $a_{0}^{*}$, $b_{0}^{*}$) were used as initial samples (reference) and low ΔE value corresponds to a low color change from the reference raisins [23,24].

(6) $$\Delta Ε=\sqrt{{\left(L_{0}^{*}-L^{*}\right)}^{2}+{\left(a_{0}^{*}-a^{*}\right)}^{2}+{\left(b_{0}^{*}-b^{*}\right)}^{2}}$$

where, ΔE: total color difference, a* = redness/greenness, b* = yellowness/blueness, L* = lightness (brightness from black (0) to white (100)).

#### 2.2.6. Visual Inspection and Microscopic Analysis of Raisin Samples

A visual inspection of the crystallized raisins was made according to USDA (U.S. Department of Agriculture) standards for processed raisins [2,25,26]. For this purpose, 400 g of raisins from each pretreatment were weighed and visual inspection was carried out in a one by one manner of the raisins under a magnifying glass. The crystallized raisins were weighed and the percentage of crystallized raisins was determined. According to USDA standards [6], a sugared sample refers to the presence of either external or internal sugar crystals and the accumulation of such crystallized fruit sugars in the flesh or on the surface of the raisins being readily (visually) apparent. Considering this classification of crystallized raisins, the size of the readily apparent crystals was determined under the advice of industrial quality control experts and corresponded to a crystal size of around 200 μm or larger.

$$\%\left(w/w\right)\;\mathrm{crystallized}\;\mathrm{raisins}\;=\;\frac{g\;\mathrm{cristallized}\;\mathrm{raisins}\;}{g\;\mathrm{total}\;\mathrm{raisins}\;\left(400\;g\right)}$$

For the microscopic analysis, samples of raisin flesh were gently spread onto the microscopic slide, following microscopic observation under a polarized light microscope (Olympus U-CTR30-2, Olympus, Shinjuku, Tokyo, Japan) at 4× magnification.

### 2.3. Thermal Pretreatment and Crystallization Kinetics of Raisins

Three thermal pretreatment conditions were analyzed. Thermal pretreatment involved thinly spreading 400 g of raisins (finished product) in aluminum trays and incubating them in a hot air oven at 50 °C and 70 °C for a period of 20 min (Biochemical Incubator; Zhongxing, City, China; Model: SHP-350; 900 W). In the microwave treatment, a Samsung (Model.M1736N, Samsung, Seoul, Korea) laboratory-scale microwave oven was used. A bed of 400 g raisins was irradiated with microwave at 800 W for 15 s.

The crystallization was monitored for a period of 35 days for the samples stored at two temperatures (15 and 25 °C) in a relative humidity environment of 57% by visual inspection and polarized microscopy (Olympus U-CTR30-2, Olympus, Shinjuku, Tokyo, Japan). The temperature and humidity conditions were selected based on the actual range of temperature and humidity likely to be realized by raisins in practice. The relative humidity (RH) condition (57% RH) was obtained using supersaturated salt solution of NaBr.

Physico-chemical analysis was carried out every 10 days. The results obtained were compared with the control samples that corresponded to the raisins without thermal pretreatment.

### 2.4. Statistical Analysis

All measurements were carried out at least in triplicate. One-way analysis of variance (ANOVA) and Fisher’s test were used to detect the difference between the mean values at *p* < 0.05 using Minitab Release 16 (Minitab LLC, State College, PA, USA).

## 3. Results and Discussion

Raisins used in this study showed a moisture content of 15.8 ± 0.5% (*w*/*w*), and a corresponding water activity of 0.586 ± 0.011.

**Figure 2:**
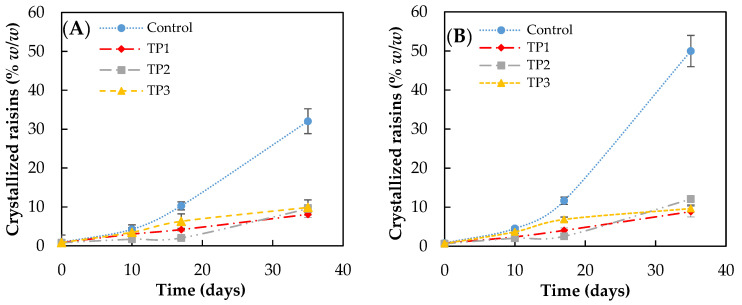
Crystallization rate of raisins (finished product) analyzed by visual inspection, stored at (**A**) 15 °C and (**B**) 25 °C and at 57% relative humidity. Control: Samples without pretreatment. TP1: Thermal pretreatment at 50 °C for 20 min. TP2: Thermal pretreatment at 70 °C for 20 min. TP3: Thermal pretreatment in microwave 800 W for 15 s.

The results of the crystallization kinetics obtained when finished products pretreatment were stored under different conditions are shown in Figure 2. The storage conditions used were 57% RH at 15 and 25 °C.

In the case of control samples, the increase in storage temperature favors crystallization (crystal growth), particularly after 20 days of storage. After 35 days of storage, the percentage of crystallized raisins incubated at 57% RH reached 32 ± 2 and 50 ± 3% (*w*/*w*) when the storage temperature was 15 and 25 °C, respectively. No significant difference was found at 35 days (*p* < 0.05) in samples with the pretreatment.

However, the thermal pretreatment of raisins caused a significant decrease in the crystallization rate after 10 days, obtaining less than 10% (*w*/*w*) of crystallized raisins after 35 days of storage at all conditions evaluated. No significant difference (*p* < 0.05) was observed between the raisins pretreated at 50 °C, 70 °C for 20 min, and microwave for a short time.

**Figure 3:**
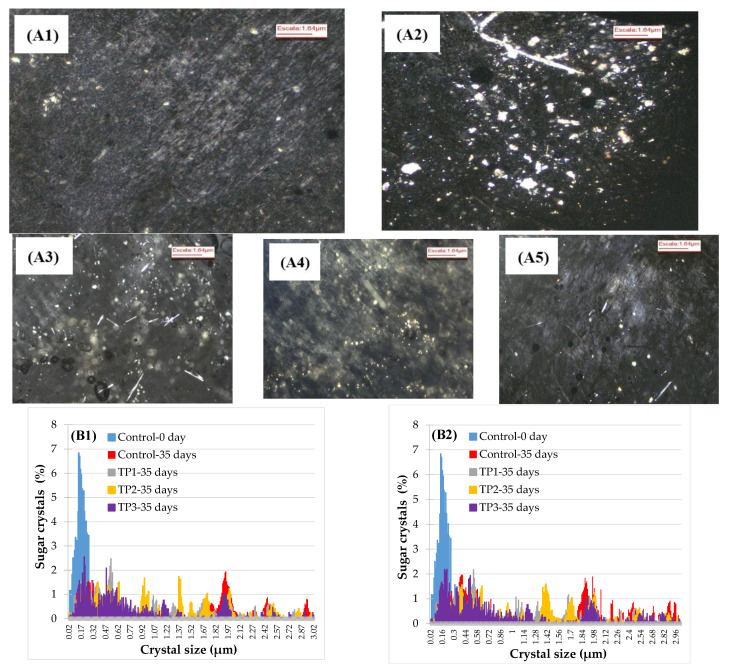
Analysis of sugar crystals size by polarized light microscopy. (**A**) Photography (4× magnification) of the control sample stored at 57% RH and 25 °C. (**A1**) initial sample, (**A2**) 35 days of sample control, (**A3**) 35 days of sample TP1, (**A4**) 35 days of sample TP2, (**A5**) 35 days of sample TP3. (**B**) The crystal size distribution of samples pretreated and stored for 35 days at 57% HR (**B1**) stored at 15 °C and (**B2**) stored at 25 °C. Control: Samples without pretreatment. TP1: Thermal pretreatment at 50 °C for 20 min. TP2: Thermal pretreatment at 70 °C for 20 min. TP3: Thermal pretreatment by microwave irradiation at 800 W for 15 s.

Samples of the raisins with and without thermal pretreatment were analyzed by polarized light microscopy (Figure 3A). The use of polarized light allowed for improving the visualization of the crystals inside the product, which appeared as a bright region surrounded by a dark background. Crystal size and distribution are presented in Figure 3B. Initially (control samples), the major part of the crystals was in the range 0.09–0.35 μm, while after 35 days of storage the size distribution changed and crystals of more substantial sizes were observed, which may be explained by the crystals’ growth. The larger size of crystals after 35 days is consistent with the kinetics obtained by visual inspection since a raisin will be classified as crystallized according to USDA standards only when crystals may be observed by the naked eye, which are in the order of around 200 μm or more in size. Our microscope examination of the surface of the raisins revealed a few scattered large individual crystals.

Bolin [12] undertook the first study to delay crystallization, demonstrating the impact of thermal pretreatment on the crystallization of raisins through visual inspection and organoleptic tests. This was attributed to the melting of pre-existing tiny crystals in the flesh which otherwise would grow larger over the time of storage. After a series of exploratory experiments where raisins were heated for 48 h at 49 °C, the author found that the thermal treatment also had an effect on the texture of the whole raisins. The author observed that, when subjected to a certain minimum heating, raisins presented a softer texture than untreated raisins. The visual inspection of the heat-treated raisins stored at 22 °C for 10 months, revealed no evidence of crystal formation. An early study by Alvarez et al. [14] also concluded that a thermal treatment for 50 °C for a period of 12 h resulted in a reduction in the percentage of crystallized raisins (*w*/*w*) from more than 46% in control samples to less than 10% after 30 days of storage.

Some of the crystals were of needle shape, very thin and fragile and broke easily. The major parts of crystals observed by the naked eye occurred on the large crease (wrinkle) on the skin surface of raisins. This could be due to a greater permeability. The surface of a raisin is composed of a network of interspersed wax platelets. Possibly, when drying, the formation of the obtuse angles of the skin may cause a stretch and separation of the wax platelets, which allows the solution of sugars to pass through the skin to the outer surface in these areas. On the surface, the rest of the water evaporates easily, leaving a saturated sugar solution that crystallizes easily in the presence of a few small seed crystals [27]. In addition, the large crystals may also pierce the weakest point and grow internally pushing the grown crystals out, appearing as white granular surface. However, microwave treatment showed the smallest sizes of crystal particle.

In order to ensure stability while in storage, the water activity (a_w_) is usually required to be lower than 0.6. At this water activity, the product undergoes limited deterioration during storage which allows the product to be made available outside of the standard harvest times without necessitating expensive continuous refrigeration or frozen storage [28]. The initial water activity and moisture content value for control samples were 0.586 and 17% (*w*/*w*), respectively. The water activity of raisins is presented in Table 1. The a_w_ of control samples increased along the 35 days of storage. The higher a_w_ after 35 days of storage may explain the higher proportion of raisins crystallized, considering that crystal growth follows a nucleation step and requires that molecules are able to diffuse to the surface of the growing nuclei [29].

Analysis of the a_w_ of thermally pretreated raisins (50 °C for 20 min, 70 °C for 20 min and microwave 800 W for 15 s) is shown in Table 1. The initial a_w_ was significantly lower than the control (*p* < 0.05). This result can be explained by the evaporation of water during the thermal process. Even though the initial a_w_ was lower, samples with thermal pretreatment showed an increment of a_w_ along the 35 days of storage, especially at 25 °C. As may be observed, the final a_w_ of pretreated samples increased, while equilibrating with the RH of the headspace conditions. The actual time for such equilibration would have been longer than 35 days as the water activity was lower than the RH/100 of the headspace. However, crystallization was significantly lower than the control at these conditions. Another explanation could be that increased crystallization results in an expulsion water and diffusion of this water in the non-crystalline matrix increases the water activity of raisins. This is a common observation reported during crystallization of honey which has a similar sugar composition to raisins [30].

The analysis of the sugars in control samples and thermally treated samples shows an increase in the glucose/fructose ratio, as the storage time increased to the different conditions (15 °C and 25 °C, at 57% RH), (Table 1). On the other hand, when comparing the thermally pretreated samples stored for 35 days to 15 °C and 57%, the RH presented a significant difference according to Fisher’s test, *p* < 0.05 concerning their stored controls under the same conditions. However, when comparing the thermally pretreated samples stored for 35 days at 25 °C and 57% RH with respect to their respective controls, no significant variation in the glucose/fructose ratio was observed. Previous X-ray diffraction analysis identified alpha-d-Glucose mono-hydrate as the main crystalline component in the control samples and some beta-d-Glucose is highly possible. In the case of the samples treated thermally, only alpha-d-Glucose monohydrate was identified without the presence of other crystalline species in previous work by Alvarez et al. [14].

**Figure 4:**
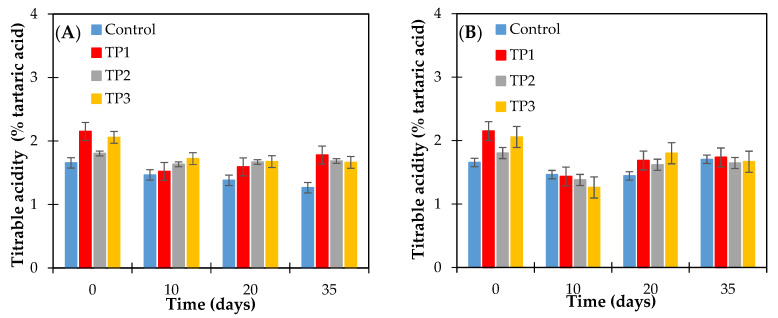
Titrable acidity content of raisins (finished product) determined as tartaric acid. Raisins were stored at 57% relative humidity and at (**A**) 15 and (**B**) 25 °C. Control: Samples without pretreatment. TP1: Thermal pretreatment at 50 °C for 20 min. TP2: Thermal pretreatment at 70 °C for 20 min. TP3: Thermal pretreatment in microwave 800 W for 15 s.

The range of acidity of the control raisins and samples thermally pretreated (50 °C and 70 °C for 20 min and microwave 800 W for 15 s) was between 1.26 ± 0.03–2.15 ± 0.06 g of tartaric acid per 100 g of dried raisins. (Figure 4). The effect of tartaric acid concentration on the crystallization of raisin sugars has not been previously reported. During the storage time at the conditions studied, a significant decrease in acidity was observed for both control and thermally treated samples, according to the Fisher test (*p* < 0.05), as shown in Figure 4. Zemni et al. Ref. [5] reports that the lowest values (1.17 g of tartaric acid/100 g of dried raisins) and highest (2.73 g/100 g of dried raisins) of titratable acidity were recorded in raisins dried in the oven and in the sun, respectively, and the decrease in titratable acidity was the result of the removal of water from the grapes. A comparable situation registered in our assays where relative humidity was controlled (at 57% or 66%).

Color parameters were investigated to evaluate the effects of thermal pretreatment of raisins to avoid crystallization of sugar at different storage conditions. Color is one of the essential criteria for the determination of quality and consumer preference of raisins [22,31,32]. Color parameters and ΔE of controls without thermal treatment, and thermal treatment (at 50 °C and 70 °C for 20 min) and thermal treatment with microwaves and incubated at different temperatures (15 °C and 25 °C) are shown in Table 2.

The results displayed in Table 2 indicate that thermal pretreatment affected all color parameters and that these were lower than in fresh raisins. The L* parameter represents lightness in terms of whiteness or brightness, and the values obtained ranged from 23.6 to 24.3 for all thermal treated and control samples with a decrease of 3 to 5% in relation to fresh raisins. This value is low compared to other studies that published L* parameters of raisins [22] and indicated that raisins were slightly darker, but still acceptable to consumers.

As for the a* parameter, all values were significantly similar to fresh raisins, indicating that raisins with or without heat treatment had the same red color. All b* parameters were higher than fresh raisins, indicating that raisins with or without thermal treatment had more yellow coloration. Tulasidas et al. [33] reported that microwave-dried grapes had a better color than the sundried ones.

Table 2 shows total color difference (ΔE) and considered as the total color difference between samples without thermal treatment (control) and with thermal treatment. In general, lower ΔE values represent fewer color changes during the thermal treatment, which make the products acceptable to the customers. The higher ΔE* was obtained for the samples with thermal treatment at 50 °C for 20 min and incubated at 15 °C, and thermal treatment at 70 °C for 20 min and incubated at 25 °C. Color is considered as the key quality index due to its relationship with flavor and aroma with dried foods being susceptible to color deterioration [34], and all samples showed a desirable color with low ΔE* value.

The texture profile analysis (TPA) data shown in Appendix A, present the results obtained from the determinations of hardness (N), springiness (%), cohesiveness, and chewiness (N) of the raisins stored for 35 days at 15 °C, 25 °C in a 57% RH environment.

Hardness refers to the force required to compress food between the teeth or between the tongue and mouth to cause deformation [22,35]. The thermal pretreatments at 70 °C for 20 min and thermal pretreatment by microwave irradiation at 800 W for 15 s increased the hardness compared with fresh raisin and control samples when they were incubated at 15 °C and 57% RH. On the other hand, the samples treated at 50 °C for 20 min and stored for 35 days at 15 °C, did not present a significant difference (*p* < 0.05) with respect to the fresh raisins (on day 0). Opposite results were reported by Alvarez et al. [14], where all thermal pretreatments increased the hardness compared with fresh raisins and controls. However, in that study, the samples were subjected to thermal treatments of 50 °C for 12 h (incubated at 25 °C) and of 50 °C for 48 h (incubated at 15 °C).

**Figure A1:**
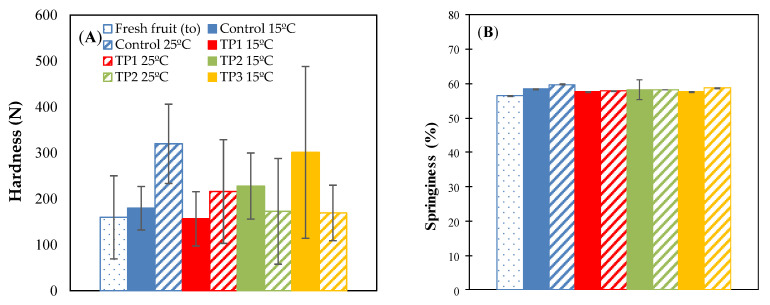

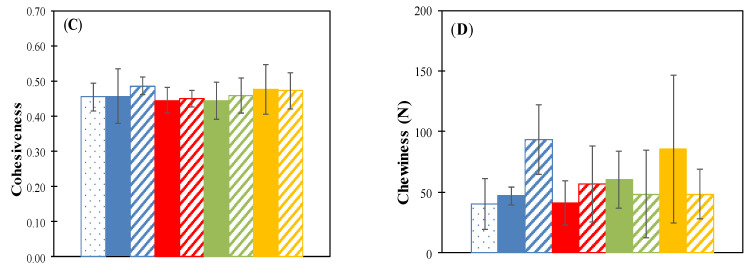
Hardness (**A**), springiness (**B**), cohesiveness (**C**) and chewiness (**D**) for different thermal pretreatment incubated at 57% RH. Control: Samples without pretreatment. TP1: Thermal pretreatment at 50 °C for 20 min. TP2: Thermal pretreatment at 70 °C for 20 min. TP3: Thermal pretreatment in microwave 800 W for 15 s.

The control samples and thermal pretreatments at 50 °C for 20 min stored at 25 °C, significantly increased in hardness in relation to fresh raisins. However, the samples treated at 70 °C for 20 min, irradiated with microwaves at 800 W for 15 s and stored at 25 °C did not show a significant difference with respect to fresh raisins (Figure A1A).

The springiness (elasticity) is the ability to recover shape after compression and measures the speed of return to the initial state after the elimination of the force that causes the deformation [22,36]. This is defined as the ratio (percentage) of the time from the start of second and first compression to the peak. The springiness values of each treatment did not show a significant difference (*p* < 0.05) compared with the fresh raisins (on day 0) and controls stored at 15 and 25 °C, 57% RH (Figure A1B). Thus, these treatments maintained the original cohesiveness (Figure A1C).

The chewiness measures the energy required to disintegrate food to be swallowed [22]. The chewiness of the control and thermally pretreated samples at 50 °C for 20 min (incubated at 15 °C) increased with respect to fresh raisins, and an increase was also observed when stored at a higher temperature (25 °C). On the other hand, the samples thermally pretreated at 70 °C for 20 min and with microwaves at 800 W for 15 s behaved in reverse, significantly reducing in chewiness as storage time increased (Figure A1D).

In the case of the microwave treatment, Carranza-Concha et al. [17] studied the use of microwave treatment of grapes and increased pectin solubilization noting a consequent change in texture and decrease in hardness compared with conventional dehydration process. A similar change might have occurred in pectin in this work. Wojdyło et al. [37] carried out a vacuum–microwave drying process to cherries. They studied what wattage must be applied to ensure optimal conditions to optimize bioactive compounds (antioxidants) and color. Zielinska et al. [19] studied the effectiveness of vacuum–microwave pretreatment on drying cranberries and determined optimal recovery of bioactive compounds. However, no published reports have been found regarding the use of microwave pretreatment to control the crystallization phenomenon in raisins.

Additionally, even if energy consumption was not the main topic of this piece of research, a comparison between both thermal pretreatments studied can be assessed. The incubator oven, 900 W power, was used to treat 400 g raisins for 20 min; while, the microwave oven, 800 W power, was used to treat 10 g of raisins for 15 s 40 times, resulting in 400 g in total. As a rough calculation, the estimated energy consumption for each thermal treatment was 0.750 kWh/kg raisins and 0.325 kWh/kg raisins, for the incubator and microwave, respectively.

## 4. Conclusions

The control of crystallization kinetics of Thompson raisins by thermal pretreatment in hot air and microwave ovens was studied at different storage conditions.

The increase in the environmental temperature from 15 °C to 25 °C favored the crystallization of the raisins without pretreatment. At higher temperatures (25 °C) the crystallization rate of control samples presented an exponential trend, with a higher than 50% sugaring rate in 35 days of storage, while the thermally treated samples presented 10% sugaring. Analysis of raisin flesh by optical polarized light microscopy showed that the crystal size distribution changed after 35 days of storage in all conditions and crystals with larger sizes were observed.

The thermal pretreatment of the samples decreased the average luminosity of the raisins, in comparison to fresh raisins, but lower ΔE (considered the total color difference between samples without thermal treatment (control) and with thermal treatment) values revealed that the products were considered acceptable for consumers. Textural parameters such as springiness and cohesiveness, were not affected by the thermal pretreatments; meanwhile, some effect on the hardness and chewiness of the control sample was observed during storage.

Power consumption estimation revealed that the microwave treatment would be significantly cheaper than thermal treatment in a conventional oven, but this must be further studied on a larger scale. Finally, combination with the heating conventional method it is not advisable since it would increase energy consumption and processing time.

## Figures and Tables

**Table 1:** Effect of storage temperatures (15 and 25 °C) in 57% RH conditions on the water activity (a_w_) and glucose/fructose ratio of the raisins with and without thermal pretreatment.

**a_w_**								
**Time (d)**	**Control**		**TP1**		**TP2**		**TP3**	
	**15 °C**	**25 °C**	**15 °C**	**25 °C**	**15 °C**	**25 °C**	**15 °C**	**25 °C**
0 ^A^	0.590 ± 0.033 ^a^	0.590 ± 0.033 ^a^	0.601 ± 0.009 ^b^	0.601 ± 0.009 ^b^	0.606 ± 0.011 ^c^	0.606 ± 0.011 ^c^	0.606 ± 0.018 ^b^	0.606 ± 0.018 ^b^
10 ^B^	0.631 ± 0.008 ^a^	0.592 ± 0.021 ^b^	0.578 ± 0.024 ^c^	0.581 ± 0.021 ^c^	0.593 ± 0.018 ^b^	0.568 ± 0.032 ^d^	0.587 ± 0.026 ^b^	0.613 ± 0.012 ^e^
20 ^B^	0.633 ± 0.015 ^a^	0.598 ± 0.012 ^b^	0.603 ± 0.010 ^c^	0.603 ± 0.008 ^c^	0.606 ± 0.013 ^c^	0.596 ± 0.020 ^b^	0.617 ± 0.012 ^d^	0.614 ± 0.022 ^d^
35 ^D^	0.643 ± 0.016 ^a^	0.599 ± 0.010 ^b^	0.596 ± 0.015 ^b^	0.608 ± 0.006 ^c^	0.615 ± 0.016 ^d^	0.609 ± 0.024 ^c^	0.622 ± 0.021 ^e^	0.620 ± 0.026 ^e^
**Glucose/Fructose Ratio**								
**Time (d)**	**Control**		**TP1**		**TP2**		**TP3**	
	**15 °C**	**25 °C**	**15 °C**	**25 °C**	**15 °C**	**25 °C**	**15 °C**	**25 °C**
0 ^A^	0.98 ± 0.08 ^a^	0.98 ± 0.09 ^a^	0.95 ± 0.07 ^b^	0.95 ± 0.09 ^b^	0.94 ± 0.12 ^c^	0.94 ± 0.10 ^c^	0.94 ± 0.12 ^c^	0.94 ± 0.08 ^c^
10 ^B^	1.01 ± 0.01 ^a^	0.99 ± 0.07 ^b^	0.93 ± 0.04 ^c^	0.98 ± 0.11 ^d^	0.96 ± 0.09 ^b^	0.96 ± 0.07 ^b^	0.98 ± 0.04 ^d^	0.96 ± 0.11 ^b^
20 ^C^	1.04 ± 0.01 ^a^	1.04 ± 0.03 ^a^	0.98 ± 0.09 ^b^	0.98 ± 0.07 ^b^	0.98 ± 0.06 ^b^	0.98 ± 0.04 ^b^	0.99 ± 0.07 ^c^	0.98 ± 0.06 ^b^
35 ^D^	1.06 ± 0.06 ^a^	1.08 ± 0.11 ^b^	1.04 ± 0.04 ^c^	1.09 ± 0.08 ^b^	1.08 ± 0.09 ^b^	1.08 ± 0.06 ^b^	1.07 ± 0.11 ^b^	1.09 ± 0.03 ^b^

TP1: Thermal pretreatment at 50 °C for 20 min.; TP2: Thermal pretreatment at 70 °C for 20 min.; TP3: Thermal pretreatment in microwave 800 W for 15 s. Different lowercase letters in same row denote a significant difference according to Fisher’s test, *p* < 0.05. Different capital letters in same column denote a significant difference according to Fisher’s test, *p* < 0.05.

**Table 2:** Color parameters of raisins without and with thermal pretreatment and stored 57% relative humidity at different conditions of temperature by 35 days.

Sample	T °C	L*	a*	b*	E*
Fresh raisin (t_o_)	n.c.	25.00 ± 2.11	4.90 ± 1.59	5.40 ± 1.74	n/a
Control-15 °C	15	23.66 ± 1.59	5.03 ± 1.34	6.12 ± 1.22	1.53 ± 0.17 ^a^
Control-25 °C	25	23.65 ± 2.03	4.71 ± 1.25	6.06 ± 1.45	1.52 ± 0.15 ^a^
TP1-15 °C	15	23.45 ± 1.48	5.19 ± 1.76	6.14 ± 1.81	1.74 ± 0.16 ^b^
TP1-25 °C	25	24.32 ± 1.58	5.34 ± 1.85	6.37 ± 1.85	1.26 ± 0.15 ^c^
TP2-15 °C	15	23.64 ± 1.84	5.01 ± 1.65	6.17 ± 1.54	1.57 ± 0.17 ^d^
TP2-25 °C	25	23.29 ± 1.28	4.53 ± 1.13	5.53 ± 1.01	1.75 ± 0.15 ^b^
TP3-15 °C	15	23.71 ± 1.91	5.21 ± 1.68	6.10 ± 1.32	1.53 ± 0.16 ^a^
TP3-25 °C	25	23.87 ± 1.12	4.65 ± 1.15	6.00 ± 1.09	1.30 ± 0.11 ^c^

Control: Samples without pretreatment. TP1: Thermal pretreatment at 50 °C for 20 min. TP2: Thermal pretreatment at 70 °C for 20 min. TP3: Thermal pretreatment by microwave irradiation at 800 W for 15 s. Different letters denote a significant difference according to Fisher‘s test, *p* < 0.05. n.c. means not controlled temperature (room temperature, approx. 18 °C). n/a: not applicable.
